# Correction: Brauner et al. Dental Management of Maxillofacial Ballistic Trauma. *J. Pers. Med.* 2022, *12*, 934

**DOI:** 10.3390/jpm13030535

**Published:** 2023-03-17

**Authors:** Edoardo Brauner, Federico Laudoni, Giulia Amelina, Marco Cantore, Matteo Armida, Andrea Bellizzi, Nicola Pranno, Francesca De Angelis, Valentino Valentini, Stefano Di Carlo

**Affiliations:** 1 Department of Oral and Maxillofacial Sciences, Sapienza University of Rome, Via Caserta 6, 00161 Rome, Italy; 2 Implanto-Prosthetic Unit, Policlinico Umberto I, Viale Regina Elena 287b, 00161 Rome, Italy; 3 Oncological and Reconstructive Maxillo-Facial Surgery Unit, Policlinico Umberto I, Viale del Policlinico 155, 00167 Rome, Italy

The authors wish to make the following correction to this paper [1] “Dental Management of Maxillofacial Ballistic Trauma”.

The study was rightly approved by the DEPARTMENT/ISTITUTIONAL REVIEW BOARD APPROVAL n. 111/2022 Prot. N. 0000867, on 18 May 2022 of Sapienza University of Rome, but in the section Material and Methods of the article and in institutional review board statement section we wrongly wrote that the article was approved by the Ethics Committee.

Sentence: “approved by the Ethics Committee of “Sapienza” University of Rome” should be replaced with “approved by the Department Review Board Approval of Sapienza University of Rome (N. 111/2022 Prot. N. 0000867, 18 May 2022)”. The authors apologize for any inconvenience caused and state that the scientific conclusions are unaffected. The original publication has also been updated.
